# Necrotic Excluded Gastric Remnant Post Gastric Bypass: A Rare Fatal Complication

**DOI:** 10.7759/cureus.11559

**Published:** 2020-11-19

**Authors:** Humaira Haider Mahin, Mina Sarofim, Christopher S Lim

**Affiliations:** 1 General Surgery, The Wollongong Hospital, Wollongong, AUS; 2 Medicine, University of New South Wales, Sydney, AUS; 3 General Surgery, Bankstown Hospital, Sydney, AUS; 4 Hepatobiliary Surgery, Box Hill Hospital, Melbourne, AUS

**Keywords:** gastric bypass surgery, excluded gastric remnant, necrosis, perforation, complication, stomach partitioning gastrojejunostomy

## Abstract

We report an unusual case of a 76-year-old woman with a necrotic perforated excluded gastric pouch who had stomach partitioning gastrojejunostomy 20 years earlier for morbid obesity. The necrotic mucosa of the excluded gastric pouch was seen on gastroscopy with retrograde cannulation from the pylorus. Laparotomy revealed a distended excluded stomach with full-thickness ischaemia of the posterior wall with perforation into the lesser sac. Partial gastrectomy with Roux-en-Y gastrojejunostomy was performed. We strongly suggest early surgical exploration for these patients when they are hemodynamically unstable or do not have a precise diagnosis despite imaging to prevent potentially life-threatening gastric pouch necrosis. We advocate for avoiding risk factors like alcohol, nicotine, and nonsteroidal anti-inflammatory drugs (NSAIDs) and implement preoperative *Helicobacter pylori* testing and its eradication to reduce the incidence of perforation in the excluded pouch.

## Introduction

The Obesity Society, Australia anticipates the adult population prevalence of normal healthy weight will decrease from 40.6% to 22.9% over the period from 2001 and 2025. Obesity in Australia will increase from 20.5% to 33.9% [1]. Bariatric surgeries are the best available means to achieve durable and meaningful weight loss. The commonly reported late complications of gastric bypass surgery are dumping syndrome (nearly 20%), anastomotic complications such as leak or strictures (21%), abdominal hernias (7%), and infections (6%) [2]. This case shows necrosis of gastric remnant occurring two decades after a gastric bypass operation.

## Case presentation

**Figure 1:**
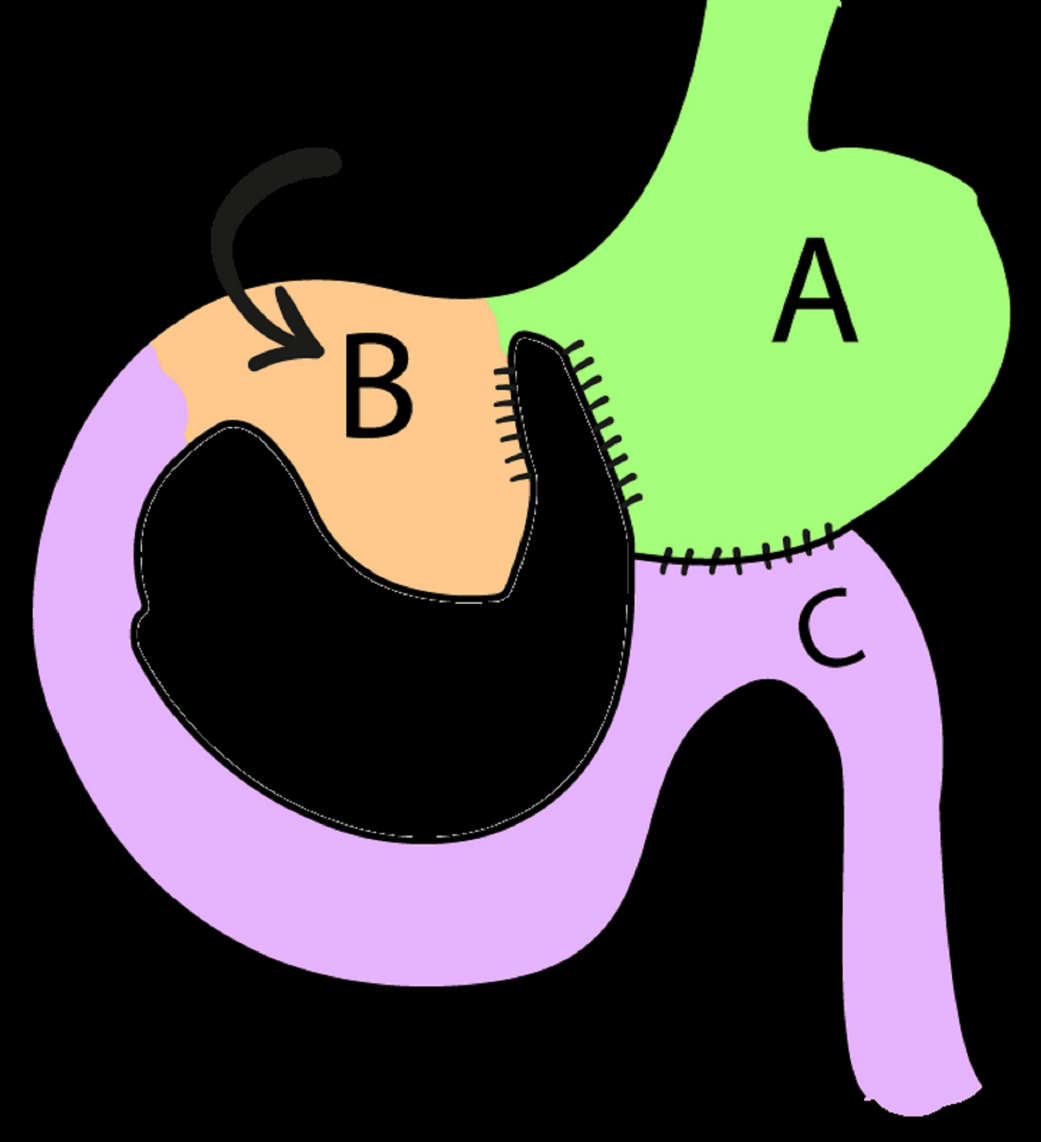
Stomach partitioning gastrojejunostomy A = partitioned stomach B = excluded gastric remnant C= jejunum, forming gastrojejunostomy

A 76-year-old female was referred to our unit for surgical management of upper gastrointestinal bleeding after unsuccessful endoscopic therapy. She had undergone a stomach partitioning gastrojejunostomy 20 years prior which made access with endoscopy difficult (Figure 1).

**Figure 2:**
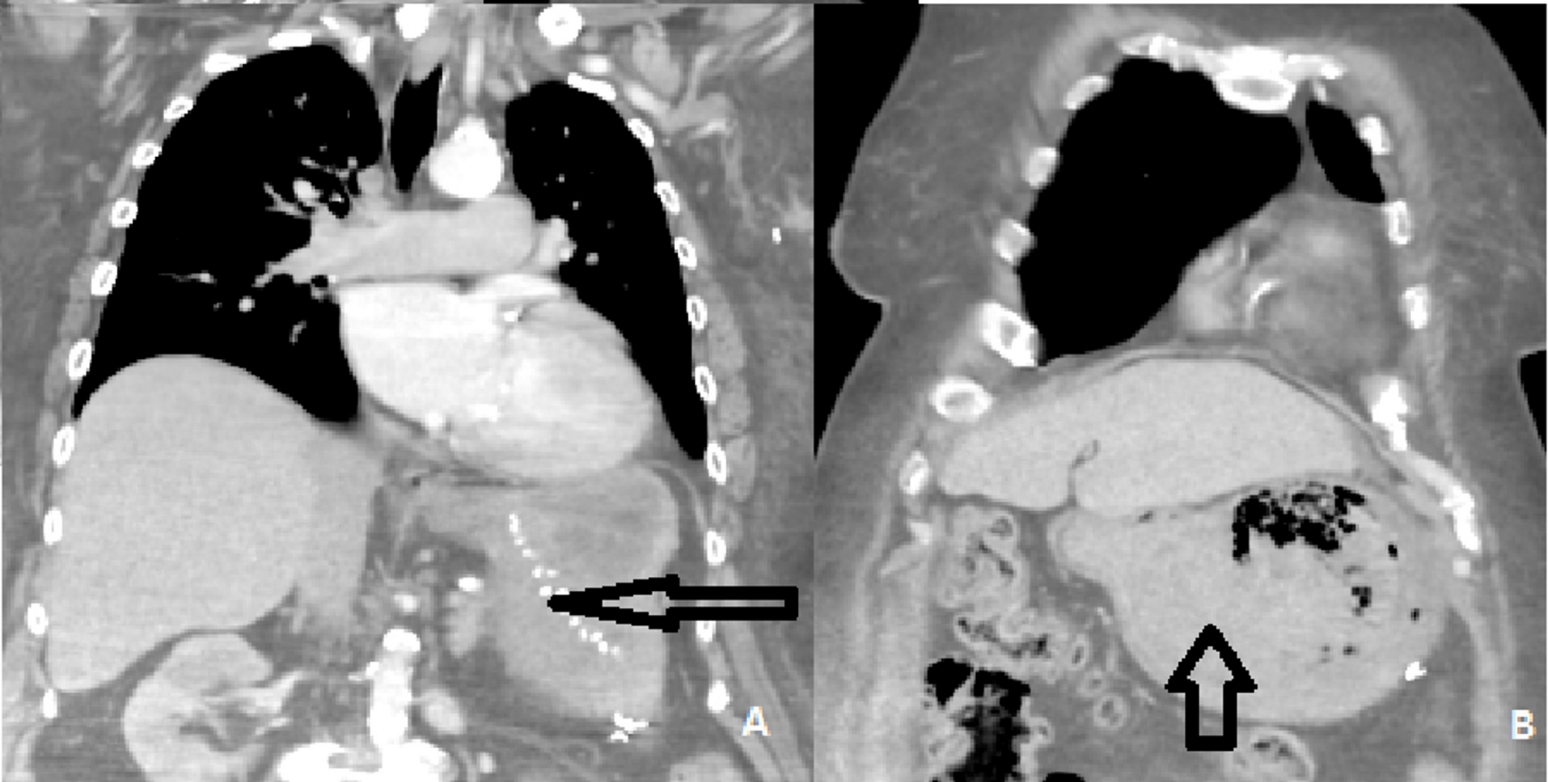
Computed tomography of chest abdomen and pelvis angiography A = arrow showing the evidence of previous gastric bypass B = arrow showing the distended stomach pouch containing blood clot

**Figure 3:**
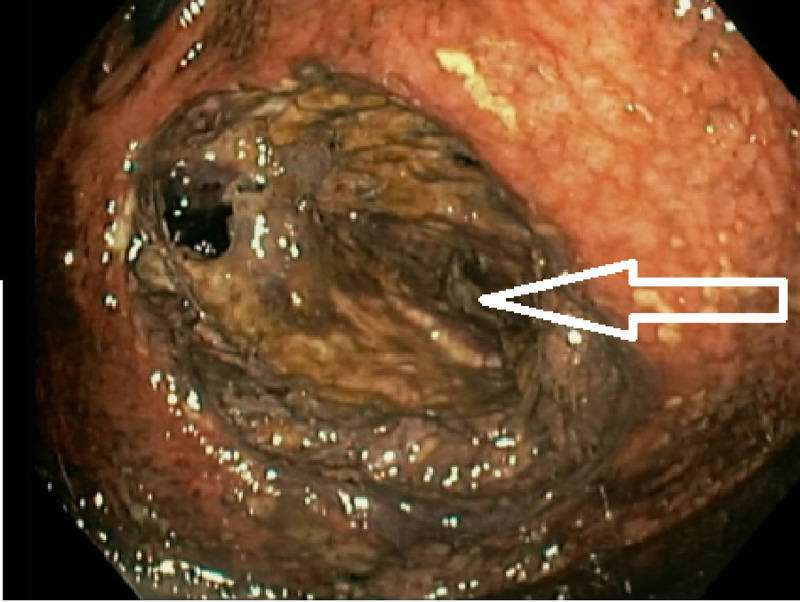
Endoscopic finding of necrotic stomach pouch White arrow showing the large necrotic cavity in the excluded stomach viewed from the pylorus from a retrograde cannulation

Relevant other medical history included morbid obesity (BMI 62.5 kg/m2), atrial fibrillation, insulin-dependent diabetes mellitus, and obstructive sleep apnoea and iron deficiency anaemia. She was a non-smoker and was not on any regular non-steroidal anti-inflammatory drugs (NSAIDs) or regular proton pump inhibitors. There was no prior history of fever, abdominal discomfort/pain or vomiting but only melena. Her vital signs were within the normal range, and her abdominal examination revealed mild tenderness in the epigastrium. Her blood results showed haemoglobin 75 g/L, white cell count of 7.32x10^9^/L, urea 14 mmol/L and creatinine 100 micromole/L. An abdominal CT scan showed mild distension of the stapled stomach, measuring up to 10 cm in diameter (Figure 2) with a mixture of gas and high-density material, suspicious for blood clot but no evidence of active bleeding. Her initial gastroscopy showed normal proximal gastric pouch but mucosal necrosis in the excluded stomach pouch adjacent to the pylorus, which was biopsied (Figure 3).

**Figure 4:**
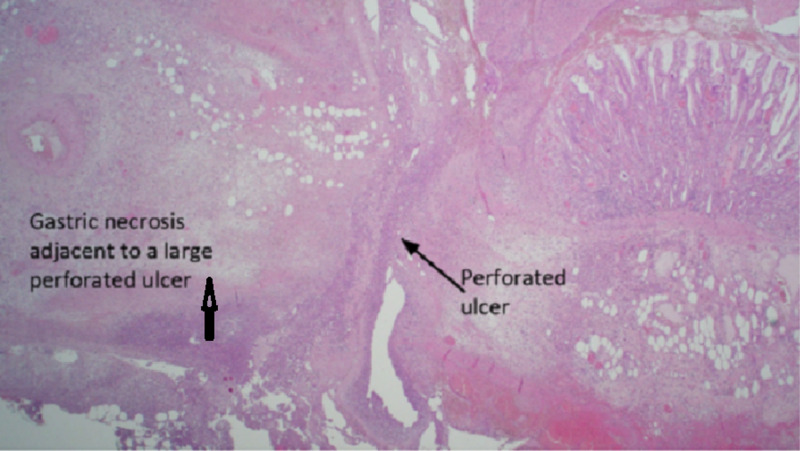
Histopathology slide Verticle arrow shows the gastric necrosis next to a large perforated ulcer

She was managed conservatively with high dose proton pump inhibitors (PPIs). However, she still experienced ongoing melena and required daily blood transfusions which prompted a surgical referral. She was taken to the operating theatre for an exploratory laparotomy which revealed a distended excluded stomach with full-thickness ischemia of the posterior wall with perforation into the lesser sac. Duodenum, jejunum and colon appeared healthy and viable. Due to concern of possible gastro-gastric fistula, the gastrojejunostomy was excised with the excluded stomach portion. A new Roux-en-Y reconstruction was performed subsequently. Histopathology showed tissue necrosis, and a dense mixed inflammatory cell infiltrates with fat necrosis and suppuration with the presence of *Helicobacter pylori* (Figure 4).

There was no evidence of neoplasia. Postoperatively, she failed to improve, developing pneumonia and intra-abdominal collections which were managed with antibiotics and percutaneous drainage. She subsequently developed multi-organ failure and unfortunately passed away three weeks after her operation.

## Discussion

According to the 2017/18 report from Bariatric Surgery Registry, the most common weight loss procedures performed in Australia are sleeve gastrectomy (84%), Roux-en-Y gastric bypass (6.2%), one anastomosis gastric bypass (4.7%), and gastric banding (4.2%) [3]. Gastric partitioning gastrojejunostomy operation is not commonly performed in Australia.

Haemorrhage after gastric bypass has been reported and bleeding can be intraluminal or intraperitoneal. Patients can present with hematemesis or melena or anaemia. Delayed bleeding may result from anastomotic erosions and ulcerations. Rapid gastrointestinal bleeding is typically identified by angiography, computed tomography (CT) scan, and upper endoscopy [2]. For our patient, CT angiography did not show any active bleeding, and retrograde gastroscopy through the pylorus showed a necrotic excluded gastric pouch without any active bleeding.

The risk factors for gastric remnant perforation and marginal ulcers are similar, and these include smoking, alcohol abuse, non-steroidal anti-inflammatory use, and *H. pylori* [4]. There is evidence that acidity may play a role in the disease pathophysiology. The buffering capacity of ingested food is absent in the gastric remnant. Cytotoxic effects of *H. pylori* and reflux of bile-containing duodenal and jejunal contents are other contributing factors for this ulceration after gastrojejunostomy [5].

In gastric bypass surgery, few risk factors may trigger necrosis: obstruction at gastrojejunostomy due to oedema, adhesions or even internal hernia causing distension and gastric or jejunal wall necrosis [6]. The reported cases of a perforated duodenal ulcer are found to be occurred within between one to 12 years after gastric bypass [6,7]. To our knowledge, there is no reported case of necrosis of a remnant gastric pouch two decades after a stomach partitioning gastrojejunostomy.

Gastric necrosis and perforation usually have a poor outcome with a recorded mortality rate of 50-80%, particularly in cases of delayed diagnosis and treatment [8]. The typical radiological sign of perforated viscus, free gas under the diaphragm, rarely occurs in the perforations of the excluded gastric remnant after gastric bypass surgery because the residual gastric pouch is unlikely to contain air.

## Conclusions

Due to this rarity, there is no guideline of management of necrosis of gastric pouch after stomach partitioning gastrojejunostomy. We performed an excision of the excluded stomach. Delayed perforations of the excluded stomach after gastric bypass are difficult to diagnose. In obese patients, the computed tomography CT scan is considered the gold standard diagnostic test. We suggest surgical exploration for hemodynamically unstable patients or for those who do not have a precise diagnosis despite imaging. We advocate for avoiding risk factors like alcohol, nicotine, and NSAIDs, and preoperative *H. pylori* testing and its eradication to reduce the incidence of perforation in the excluded pouch.
